# Structural and virologic mechanism of the emergence of resistance to M^pro^ inhibitors in SARS-CoV-2

**DOI:** 10.1073/pnas.2404175121

**Published:** 2024-09-05

**Authors:** Shin-ichiro Hattori, Haydar Bulut, Hironori Hayashi, Naoki Kishimoto, Nobutoki Takamune, Kazuya Hasegawa, Yuri Furusawa, Seiya Yamayoshi, Kazutaka Murayama, Hirokazu Tamamura, Mi Li, Alexander Wlodawer, Yoshihiro Kawaoka, Shogo Misumi, Hiroaki Mitsuya

**Affiliations:** ^a^Department of Refractory Viral Diseases, National Center for Global Health and Medicine Research Institute, Tokyo 162-8655, Japan; ^b^Experimental Retrovirology Section, HIV and AIDS Malignancy Branch, National Cancer Institute, NIH, Bethesda, MD 20892; ^c^Division of Infectious Diseases, International Research Institute of Disaster Science, Tohoku University, Miyagi 980-8575, Japan; ^d^Department of Environmental and Molecular Health Sciences, Faculty of Life Sciences, Kumamoto University, Kumamoto 862-0973, Japan; ^e^Structural Biology Division, Japan Synchrotron Radiation Research Institute, Hyogo 679-5198, Japan; ^f^Division of Virology, Institute of Medical Science, University of Tokyo, Tokyo 108-8639, Japan; ^g^The Research Center for Global Viral Diseases, National Center for Global Health and Medicine Research Institute, Tokyo 162-8655, Japan; ^h^International Research Center for Infectious Diseases, Institute of Medical Science, University of Tokyo, Tokyo 108-8639, Japan; ^i^Division of Biomedical Measurements and Diagnostics, Graduate School of Biomedical Engineering, Tohoku University, Miyagi 980-8575, Japan; ^j^Department of Medicinal Chemistry, Institute of Biomaterials and Bioengineering, Tokyo Medical and Dental University, Tokyo 101-0062, Japan; ^k^Center for Structural Biology, National Cancer Institute, Frederick, MD 21702; ^l^Basic Science Program, Frederick National Laboratory for Cancer Research, Frederick, MD 21702; ^m^Department of Pathobiological Sciences, School of Veterinary Medicine, University of Wisconsin–Madison, Madison, WI 53711; ^n^Department of Clinical Sciences, Kumamoto University Hospital, Kumamoto 860-8556, Japan

**Keywords:** SARS-CoV-2, main protease, drug resistance

## Abstract

We generated SARS-CoV-2 variants resistant to three SARS-CoV-2 main protease (M^pro^) inhibitors (nirmatrelvir, TKB245, and 5h), by propagating the ancestral SARS-CoV-2^WK521^_WT_ in VeroE6^TMPRSS2^ cells with increasing concentrations of each inhibitor and examined their structural and virologic profiles. A predominant E166V-carrying variant (SARS-CoV-2^WK521^_E166V_), which emerged when passaged with nirmatrelvir and TKB245, proved to be resistant to the two inhibitors. A recombinant SARS-CoV-2_E166V_ was resistant to nirmatrelvir and TKB245, but sensitive to 5h. X-ray structural study showed that the dimerization of M^pro^ was severely hindered by E166V substitution due to the disruption of the presumed dimerization-initiating Ser1’-Glu166 interactions. TKB245 stayed bound to M^pro^_E166V_, whereas nirmatrelvir failed. Native mass spectrometry confirmed that nirmatrelvir and TKB245 promoted the dimerization of M^pro^, and compromised the enzymatic activity; the Ki values of recombinant M^pro^_E166V_ for nirmatrelvir and TKB245 were 117±3 and 17.1±1.9 µM, respectively, indicating that TKB245 has a greater (by a factor of 6.8) binding affinity to M^pro^_E166V_ than nirmatrelvir. SARS-CoV-2^WK521^_WT_ selected with 5h acquired A191T substitution in M^pro^ (SARS-CoV-2^WK521^_A191T_) and better replicated in the presence of 5h, than SARS-CoV-2^WK521^_WT_. However, no significant enzymatic or structural changes in M^pro^_A191T_ were observed. The replicability of SARS-CoV-2^WK521^_E166V_ proved to be compromised compared to SARS-CoV-2^WK521^_WT_ but predominated over SARS-CoV-2^WK521^_WT_ in the presence of nirmatrelvir. The replicability of SARS-CoV-2^WK521^_A191T_ surpassed that of SARS-CoV-2^WK521^_WT_ in the absence of 5h, confirming that A191T confers enhanced viral fitness. The present data should shed light on the understanding of the mechanism of SARS-CoV-2’s drug resistance acquisition and the development of resistance-repellant COVID-19 therapeutics.

## Significance

The present X-ray crystallographic and virologic study demonstrates how SARS-CoV-2's main protease (M^pro^) inhibitor resistant variants emerge when SARS-CoV-2 is propagated in VeroE6^TMPRSS2^ cells under inhibitors’ selection pressure. We found that E166V substitution acquired in M^pro^ (M^pro^_E166V_) disrupts the presumed M^pro^-protomer-dimerization-initiating Ser1’-Glu166 interactions, loosens nirmatrelvir’s binding to M^pro^’s active site, and compromises nirmatrelvir’s anti-SARS-CoV-2 activity. However, TKB245, a fluorinated-benzothiazole-containing M^pro^ inhibitor, stays bound to M^pro^_E166V_’s active site and exerts substantial activity against M^pro^_E166V_-carrying SARS-CoV-2, while that activity is less than that against the wild-type virus. When propagated with another M^pro^ inhibitor (5h), SARS-CoV-2 acquired A191T, became resistant to 5h, and gained improved replicability. The data shed light on developing more potent agents against SARS-CoV-2 strains including drug-resistant variants.

Coronavirus disease 2019 (COVID-19), caused by the severe acute respiratory syndrome virus 2 (SARS-CoV-2, SCoV2), has been one of the most devastating pandemics of recent times (1, 2). More serious global health crises would have occurred if the lack of antivirals against COVID-19 continued (2–4). However, the emergence and approval of potent inhibitors of the viral main protease (M^pro^), such as nirmatrelvir (5, 6), offered hope not only on the therapeutic front but also in the context of prophylaxis against severe COVID-19. By their nature, RNA viruses including HIV inherently have high mutation rates, and lessons learned from previous and currently ongoing pandemics have taught us that these viruses can promptly develop drug resistance through mutations of vital target amino acid residues, although the actual clinical contributions of such phenotypic SCoV2 resistance against M^pro^ inhibitors (M^P^Is) remain to be studied.

Successful antiviral drugs, in theory, exert their virus-specific effects by interacting with viral receptors, virally encoded enzymes, viral structural components, or their transcripts without disturbing cellular metabolism or functions. Presently, four antiviral drugs have been approved for the treatment of SARS-CoV-2 infection, including two RNA-dependent RNA-polymerase (RdRp) inhibitors, remdesivir and molnupiravir, and two main protease (M^pro^) inhibitors, nirmatrelvir/ritonavir, and ensitrelvir. These medications have been used extensively for nonhospitalized and hospitalized patients without apparent development of widespread antiviral resistance (7, 8). Nevertheless, there have been various reports of the emergence of drug resistance to remdesivir, in particular, in immunocompromised patients (9–11). Cell culture experiments consisting serial transfers have also demonstrated the emergence of mutations that confer resistance to nirmatrelvir (12). The drug resistance–associated amino acid substitution(s) also represents a naturally occurring mutation and E166 is apparently a hotspot for drug resistance (7, 13).

Nirmatrelvir, an anti-SCoV2-M^pro^ agent, which has been first approved as an emergency use authorization drug and has been used as a first-line drug for treatment of COVID-19 (6). However, it has been reported that a variety of amino acid substitutions in M^pro^ have been observed in those receiving the 5-d treatment regimen of nirmatrelvir combined with ritonavir (RTV), which might prove to be associated with the emergence of drug resistance against M^P^Is (14). Indeed, there is a body of literature describing the emergence of SCoV2 variants with reduced susceptibility to M^P^Is with which SCoV2 was selected in the presence of anti-COVID-19 agents in test tubes (7, 12, 13, 15–17). However, the mechanism as to how such M^P^I-resistant SCoV2 variants emerge and function differently from the wild-type SCoV2 remains elusive. Structural profiles of such M^P^I-resistant SCoV2 variants also are not well understood.

In the present study, we demonstrate that i) different functional features caused by the amino acid mutations are seen under the selective pressure of nirmatrelvir, our anti-SCoV2 compound, TKB245 (18) or 5h (19), that ii) M^pro^_E166V_, which does not dimerize readily, dimerizes in the presence of each compound and such dimerization features are associated with the specific binding of each inhibitor to the enzyme, that iii) the unique fluorine-containing TKB245 exerts substantial activity against SCoV2_E166V_ compared to nirmatrelvir although the activity of TKB245 was rather attenuated than against SCoV2_WT,_ and that iv) SCoV2 variants examined here have different replicability features as precisely determined in the exquisite competitive SCoV2 replication assay (CSRA).

## Results

### SCoV2 Acquires Resistance to M^P^Is When Passaged in the Presence of Increasing Concentrations of M^P^Is.

We first determined the antiviral activity of three M^P^Is (nirmatrelvir, TKB245, and 5h) (6, 18–20) in two target cell populations, Vero E6^TMPRSS2^ cells and Hela^hACE2/TMPRSS2^ cells. As we previously described (18), TKB245 proved to be most potent against the ancestral SCoV2 strain Japan/TY/WK-521/2020 (SCoV2^WK521^) among three compounds with EC_50_ values of 0.33 µM in Vero E6^TMPRSS2^ cells and 0.0015 µM in Hela^hACE2/TMPRSS2^ cells (*SI Appendix*, Table S1). The antiviral potency of nirmatrelvir and 5h followed that of TKB245 (*SI Appendix*, Table S1).

**Fig. 1.**
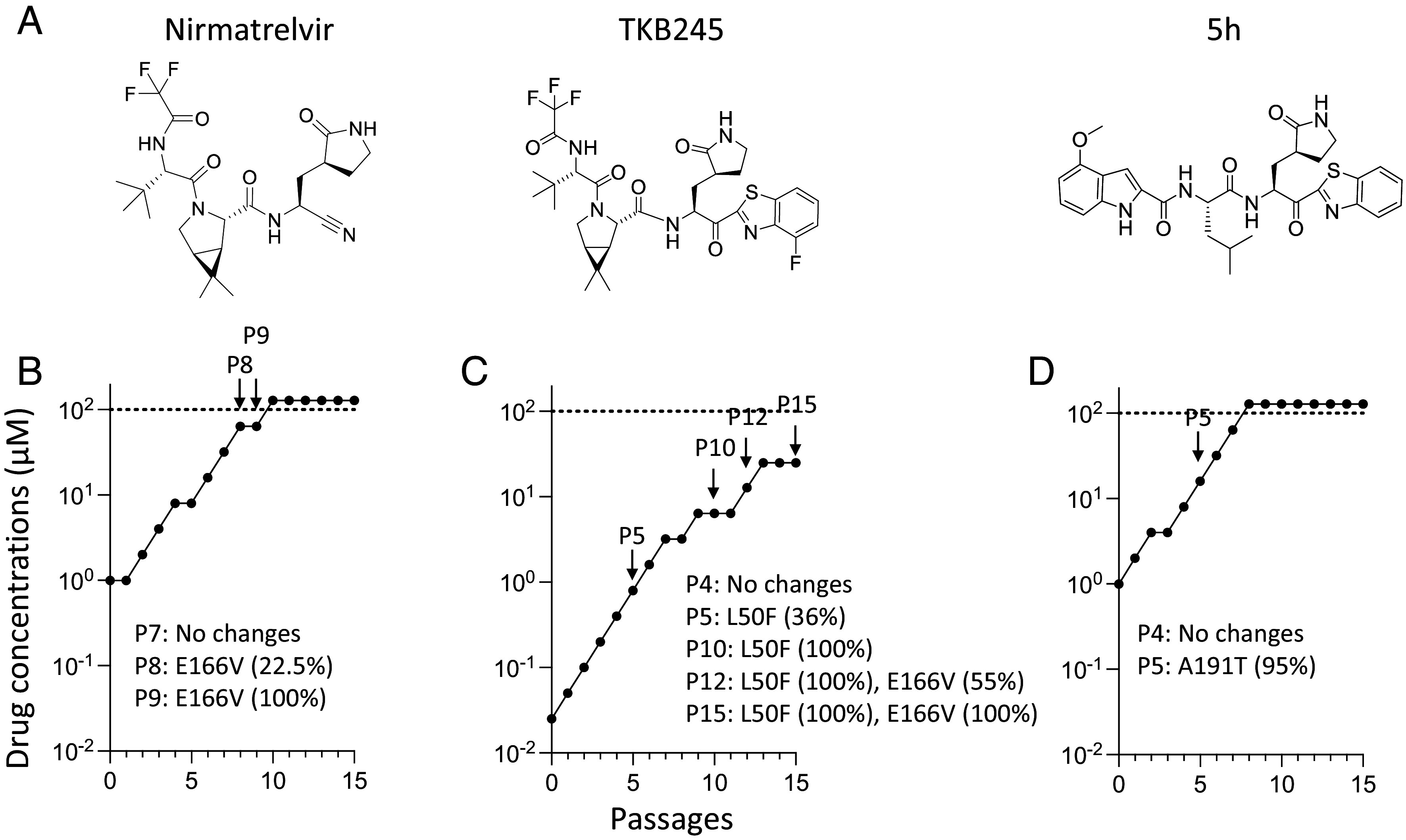
Passage-dose curve for selection of drug-resistant SARS-CoV-2 variants. Structures of compound in this study are shown (*A*). Selection was initiated concentrations at 1.0, 0.025, and 1.0 µM of nirmatrelvir (*B*), TKB245 (*C*), and 5h (*D*), respectively. At every passage, the ensuing new cell cultures were extended with the same concentration as well as a lower and a higher concentration of each compound. Arrows denote the passage numbers where amino acid substitution(s) was identified with the Sanger sequencing method. The P of P7 etc. denotes “passage.”

In order to obtain SCoV2 variants with reduced susceptibility against M^P^Is, we continuously propagated SCoV2^WK521^ in the presence of increasing concentrations of nirmatrelvir, TKB245, or 5h (Fig. 1*A*) in VeroE6^TMPRSS2^ cells. VeroE6^TMPRSS2^ cells are highly susceptible to the infectivity of SCoV2^WK521^ and well support SCoV2^WK521^ replication (19, 21). The selection was started at a 0.2-fold EC_50_ dose of each compound, and for the next-round passage, the virus was cultured at the same concentration and at concentration twice higher. When the culture demonstrates virus breakthrough that is observed as a significant cytopathic effect in the target cells, the higher concentration was used for the next passage. In the present passaging culture method, it was possible to quickly increase nirmatrelvir concentrations and reach 100 µM by 15 passages. At that stage we identified the emergence of two amino acid substitutions: L50F and E166V (Fig. 1*B* and *SI Appendix*, Fig. S1*A*)

Since the antiviral potency of TKB245 was greater than that of nirmatrelvir (*SI Appendix*, Table S1), a low concentration of TKB245 (0.025 µM) was used as an initial selection concentration, while the selection with nirmatrelvir was started at a higher concentration, 1 µM. We duly increased TKB245 concentration and when the concentration of TKB245 reached 6.4 µM (Fig. 1*C*, shown by a black arrowhead), an amino acid substitution L50F was identified and when TKB245 reached 25 µM at passage 15, another amino acid substitution E166V was found (Fig. 1*C* and *SI Appendix*, Fig. S1*B*). Since the replication rate decreased around passage 15, the selection procedure with TKB245 was terminated.

### The Genetic Nature of SCoV2^WK521^_E166V_ Obtained by the Selection with Nirmatrelvir.

We then determined the genetic natures of the primary SCoV2^WK521^ that was used as the starting viral population and SCoV2^WK521^_E166V_ obtained by the selection with nirmatrelvir. Viral RNA was extracted from the culture supernatants of SCoV2^WK521^ and nirmatrelvir-10-passaged SCoV2^WK521^ (SCoV2^WK521^_NIR-P10_) and were subjected to deep sequencing. The data obtained were assembled and the percentages of the virus with specific amino acid substitutions were determined. Notably, the percentage of the E166V substitution in M^pro^ was approximately 100%, strongly suggesting that the selection by nirmatrelvir served as a driver to substitute one of the critical amino acid, glutamic acid, in the center of the activity site of M^pro^ (*SI Appendix*, Fig. S2). It was also noted that an amino acid substitution, S1314G, had occurred in nsp3 encoding PL^pro^, another SCoV2 protease, at a high percentage (~100%) in the viral population; however, the position 1314 is apparently not associated with the enzymatic activity of PL^pro^ and no further study of its substitution was done in the present study (*SI Appendix*, Fig. S2). Also, six more substitutions were identified, but all of those had occurred in largely unspecified nonstructural proteins and no further studies were done (*SI Appendix*, Fig. S2).

### TKB245 Exerts Substantial Activity against Nirmatrelvir-Selected SCoV2^WK521^_E166V_ Compared to Nirmatrelvir.

We next examined whether the susceptibility of the SCoV2 variants selected with NIR and TKB245 was altered in the cell-based viral suppression assays using VeroE6^TMPRSS2^ cells as target cells. As shown in *SI Appendix*, Table S1, the EC_50_ values of NIR and TKB245 against SCoV2^WK521^_WT_ were 1.5 and 0.33 µM, respectively; however, those against SCoV2^WK521^_E166V_ proved to be much greater, >100 and 13 µM, respectively in Vero E6^TMPRSS2^ cells. Since VeroE6 cells are known to express a large amount of P-glycoprotein 1 (P-gp) known to pump foreign substances out of cells (22, 23) and the EC_50_ values tend to be high, we also used Hela^hACE2/TMPRSS2^ cells as targets. As expected, the EC_50_ values of NIR and TKB245 against SCoV2^WK521^_WT_ were much lower with 0.052 and 0.0015 µM, respectively. The difference in the two values was substantial by 34.7-fold. However, those against SCoV2^WK521^_E166V_ proved to be greater with 29 and 0.14 µM, respectively. The difference in the two values was 207-fold. To further examine whether the E166V substitution in the enzyme M^pro^ was responsible for the apparent resistance acquired against NIR and TKB245, we obtained two recombinant enzymes, M^pro^_WT_ and M^pro^_E166V_. As shown in *SI Appendix*, Table S2, while the k_cat_/K_m_ ratio of M^pro^_WT_ was 286.55, that of M^pro^_E166V_ turned out to be 2.397, indicating that the enzymatic activity altered by the E166V substitution was associated with the viral decreased susceptibility changes seen in the cell-based assay. As shown in *SI Appendix*, Table S1, the Ki values for nirmatrelvir and TKB245 are 117 ± 3 and 17.1 ± 1.9 µM, respectively, demonstrating that TKB245 has a greater (by a factor of 6.8) binding affinity to M^pro^_E166V_ than nirmatrelvir. With these data together, we should be able to say more specifically that the enzymatic activity altered by the E166V substitution is associated with the decreased viral susceptibility seen in the cell-based assay.

We also generated recombinant SCoV2 by using reverse genetics (rgSCoV2) (22) and determined the antiviral activity of the three M^P^Is (nirmatrelvir, TKB245, and 5h) in VeroE6^TMPRSS2^ cells (*SI Appendix*, Table S3). TKB245 showed the most potent activity against rgSCoV2 carrying wild-type M^pro^ (rgSCoV2_WT_) with an EC_50_ value of 0.78 µM, followed by nirmatrelvir and 5h with EC_50_ values of 4.4 and 13.5 µM, respectively, consistent with the results of the experiments using the ancestral strain, SCoV2^WK521^_WT_ (*SI Appendix*, Tables S1 and S3). Another rgSCoV2 carrying the E166V substitution obtained through reverse genetics (rgSCoV2_E166V_) was resistant to nirmatrelvir and TKB245 with EC_50_ values of >100 and 6.6 µM, respectively, comparable to the nirmatrelvir -selected virus populations (SCoV2^WK521^_E166V_) (*SI Appendix*, Tables S1 and S3). To examine the effect of the L50F substitution in SCoV2^WK521^_E166V_, we also generated rgSCoV2_L50F/E166V_, which showed resistance to nirmatrelvir and TKB245 with EC_50_ values of >100 μM and 11.4 μM, respectively (*SI Appendix*, Table S3). Iketani and his group also observed that the decreased susceptibility of rgSCoV2_E166V_ to nirmatrelvir and that of rgSCoV2_L50F/E166V_ were comparable (12). In fact, as examined using the X-ray structural analysis as shown in *SI Appendix*, Fig. S3, the addition of L50F substitution to M^pro^_E166V_ was seen not to affect the size or shape of M^pro^’s binding pocket when in complex with nirmatrelvir. Therefore, we assumed that the L50F substitution is important to the replicative fitness of the virus. Accordingly, we further focused on elucidating the structural and virologic nature of the E166V substitution.

### E166V Substitution Structurally Disrupts Ser1’ and Glu166 Interactions in M^pro^.

**Fig. 2.**
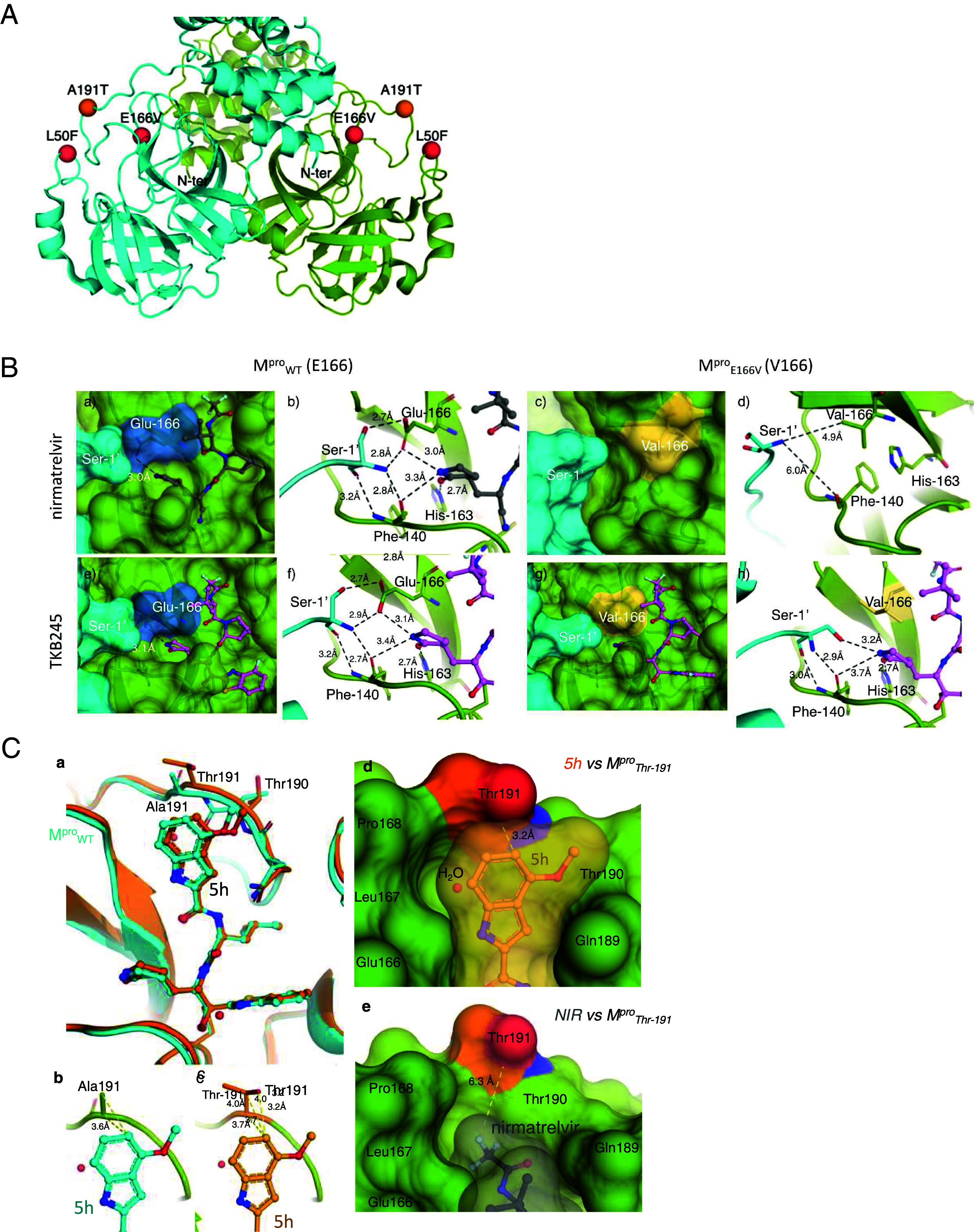
X-ray crystal structures of M^pro^_WT_, M^pro^_E166V_, and M^pro^_A191T_ in complex with nirmatrelvir, TKB245, or 5h. (*A*) The locations of amino acid substitutions that emerged as selected with nirmatrelvir, TKB245, and 5h. E166V and L50F shown as red spheres emerged as selected with nirmatrelvir and TKB245. A191T shown as orange spheres emerged as selected with 5h. (*B*) Comparison of the X-ray structures of M^pro^_WT_ and M^pro^_E166V_ in complex with nirmatrelvir (in gray sticks) and TKB245 (in pink sticks). *Insets* (*a* and c) depict the structures of M^pro^_WT_ (surface representation) bound to nirmatrelvir and the ligand-free M^pro^_E166V_, respectively. The hydrophobic side chain of V166 in M^pro^_E166V_ at least in part disrupts the inhibitor's binding, resulting in the loss of a crucial hydrogen bond between E166 and the amino group of the γ-lactam ring of nirmatrelvir. *Insets* (*b* and *d*) illustrate the hydrogen bond interaction network (black dashed lines) in M^pro^_WT_ and M^pro^_E166V_, respectively (cartoon representation). *Insets* (*e* and *g*) depict the crystal structures of TKB245 bound to M^pro^_WT_ (surface representation) or M^pro^_E166V_ (yellow), respectively. The hydrophobic side-chain of V166 in M^pro^_E166V_ slightly disrupts the binding with TKB245, resulting in the loss of a crucial hydrogen bond between E166 and the amino group of the γ-lactam ring. *Insets* (*f* and *h*) illustrate the hydrogen bond interactions (black dashed lines) in M^pro^_WT_ and M^pro^_E166V_, respectively (cartoon representation). Unlike when M^pro^_E166V_ was attempted to get crystalized with nirmatrelvir, the γ-lactam of TKB245 remained to interact with His-163 (2.8 Å) of M^pro^_E166V_, and was readily complexed with M^pro^_E166V_ [*Insets* (*g* and *h*)]. (*C*) Structural comparison of 5h binding to M^pro^_WT_ and M^pro^_A191T_. *Inset* (*a*) depicts a superposition of the crystal structures of 5h bound to M^pro^_WT_ (cyan) and M^pro^_A191T_ (orange). *Insets* (*b* and *c*): Yellow dashed lines indicate the distances between 5h and Ala191 or Thr191. In M^pro^_WT_, the distal ring of the indole moiety of 5h interacts with Ala191 at a distance of 3.6 Å. However, in M^pro^_A191T_-5h complex, these interactions are disrupted, pushing the 5h molecule further away. The structures of M^pro^_A191T_ complexed with 5h or nirmatrelvir are shown in green, with the Thr191 residue highlighted with carbon in orange, oxygen in red, and nitrogen in blue [*Insets* (*d* and *e*)]. While 5h molecule forms close contact with Thr191, nirmatrelvir's trifluoroacetyl moiety is smaller and inserted into the S4 subsite, replacing the water molecule seen in the 5h-M^pro^_A191T_ complex [*Insets* (*d* and *e*)]. As a result, the trifluoroacetyl group of nirmatrelvir is positioned further away (~6.3 Å) compared to 5h's indole ring (~4.0 Å), resulting in less anti-viral potency of 5h with M^pro^_A191T_. The increased distance is likely due to the repulsive electrostatic interaction between the hydroxyl group of threonine and the distal edge of 5h’s indole moiety [*Inset* (*d*)]. Such disruption does not occur as nirmatrelvir interacts with threonine [*Inset* (*e*)].

In an attempt to examine the structural mechanism(s) of the decreased susceptibility to nirmatrelvir brought about by E166V substitution, we conducted X-ray crystallographic analyses on M^pro^ complexed with nirmatrelvir or TKB245. Fig. 2*A* illustrates the locations of E166V and L50F substitutions in M^pro^ that occurred as SCoV2^WK521^_WT_ was selected with nirmatrelvir (Fig. 1). As featured in Fig. 2 *B*, *a* and *b*, in M^pro^_WT_ complexed with nirmatrelvir Glu166 and Phe140 formed substantial interactions with Ser1’ of another protomer with the distances ranging 2.7 to 3.2 Å. Considering that Ser1’ most likely plays critical roles in the initiation and/or progression of the dimerization process that is needed for the acquisition of the enzymatic function of M^pro^, it is thought that the disruption of the interactions between Ser1’ and Glu166 substantially reduces the enzymatic activity (24, 25). Notably, the γ-lactam moiety of nirmatrelvir formed a tight hydrogen bond network with three M^pro^ amino acids, Glu166, Phe140, and His163 with the distances ranging 2.7 to 3.3 Å (Fig. 2 *B*, *b*). However, when we attempted to crystalize M^pro^_E166V_ with nirmatrelvir under the same conditions used for M^pro^_WT_, nirmatrelvir was not seen to complex with M^pro^_E166V_ (Fig. 2 *B*, *c* and *d*). Instead, the structural changes that occurred with E166V substitution showed that the interactions between Ser1’ and Val166, and between Ser1’ and Phe140 were lost (6.7 and 7.1 Å or 4.9 and 6.0 Å, respectively) (Fig. 2 *B*, *d*). It is noteworthy that the tight interactions between the γ-lactam moiety of TKB245 and Glu166 and His163 were seen in the complex of M^pro^_WT_ and TKB245 (Fig. 2 *B*, *e* and *f*) and that even with the substituted Val166, the γ-lactam interaction with His163 remained unchanged (Fig. 2 *B*, *g* and *h*). These data suggest that the disruption by E166V substitution of the strong hydrogen network together with the loss of nirmatrelvir’s γ-lactam interactions with the enzyme caused the failure of nirmatrelvir’s binding to M^pro^_E166V_. However, TKB245 kept the γ-lactam binding to His163 and Phe140 of M^pro^_E166V_ and therefore, TKB245 binds to M^pro^_E166V_ and exerts more potent activity against SCoV2^WK521^_E166V_ compared to nirmatrelvir (*SI Appendix*, Table S1)

### Generation of 5h-Resistant SCoV2 Variants by Propagating SCoV2^WK521^ with 5h.

We also attempted to obtain SCoV2 variants resistant to 5h, a prototype M^P^I for TKB245 by propagating SCoV2^WK521^_WT_ in the presence of increasing concentrations of 5h (Fig. 1*D*). The 5h-selected SCoV2^WK521^_WT_ population displayed the acquisition of A191T as early as passage 5 (Fig. 1*D* and *SI Appendix*, Fig. S1*C*). This viral population, SCoV2^WK521^_A191T_, proved to be resistant to 5h: The EC_50_ value of 5h against SCoV2^WK521^ was 4.1 µM, while that against SCoV2_A191T_ was >100 µM (*SI Appendix*, Table S1). Interestingly, SCoV2^WK521^_A191T_ remained susceptible to TKB245 (EC_50_ = 5.4 µM) (*SI Appendix*, Table S1). The k_cat_/K_m_ ratios of two newly generated recombinant enzymes, M^pro^_WT_ and M^pro^_A191T_, were largely comparable (286.55 and 115.04, respectively) (*SI Appendix*, Table S2), suggesting that the A191T substitution does not significantly alter the M^pro^ enzymatic activity. Considering that SCoV2_A191T_ was substantially resistant to 5h with the largely robust enzymatic activity, it was thought that the A191T substitution is associated with functions such as fitness improvement.

We thus attempted to elucidate structural changes potentially caused by the A191T substitution in M^pro^ using X-ray crystallography. As shown in Fig. 2*C*, 5h readily formed a complex not only with M^pro^_WT,_ but also with M^pro^_A191T_. In the superimposed structures (Fig. 2 *C*, *a*), both enzymes exhibit an identical binding mode, with slight deviation of the loop region around the indole moiety caused by A191T substitution. In M^pro^_WT_, the distal ring of the indole moiety of 5h interacts with the α-carbon of Ala191 at a distance of 3.6 Å (Fig. 2 *C*, *b*) and in the A191T variant, this distance slightly increased to 3.7 Å (Fig. 2 *C*, *c*). The shortest distance of 3.2 Å was measured between the side chain of the hydroxyl group of Thr191 and the distal edge of the indole ring of 5h molecule. Although this suggests anion-π interactions between the hydroxyl group of Thr191 and the distal edge of the indole moiety, the side chain of Thr191 might sterically hinder the entry of the indole ring.

### The Modes of Binding of Nirmatrelvir, TKB245, and 5h to M^pro^_WT_, M^pro^_E166V_, and M^pro^_A191T_.

**Fig. 3.**
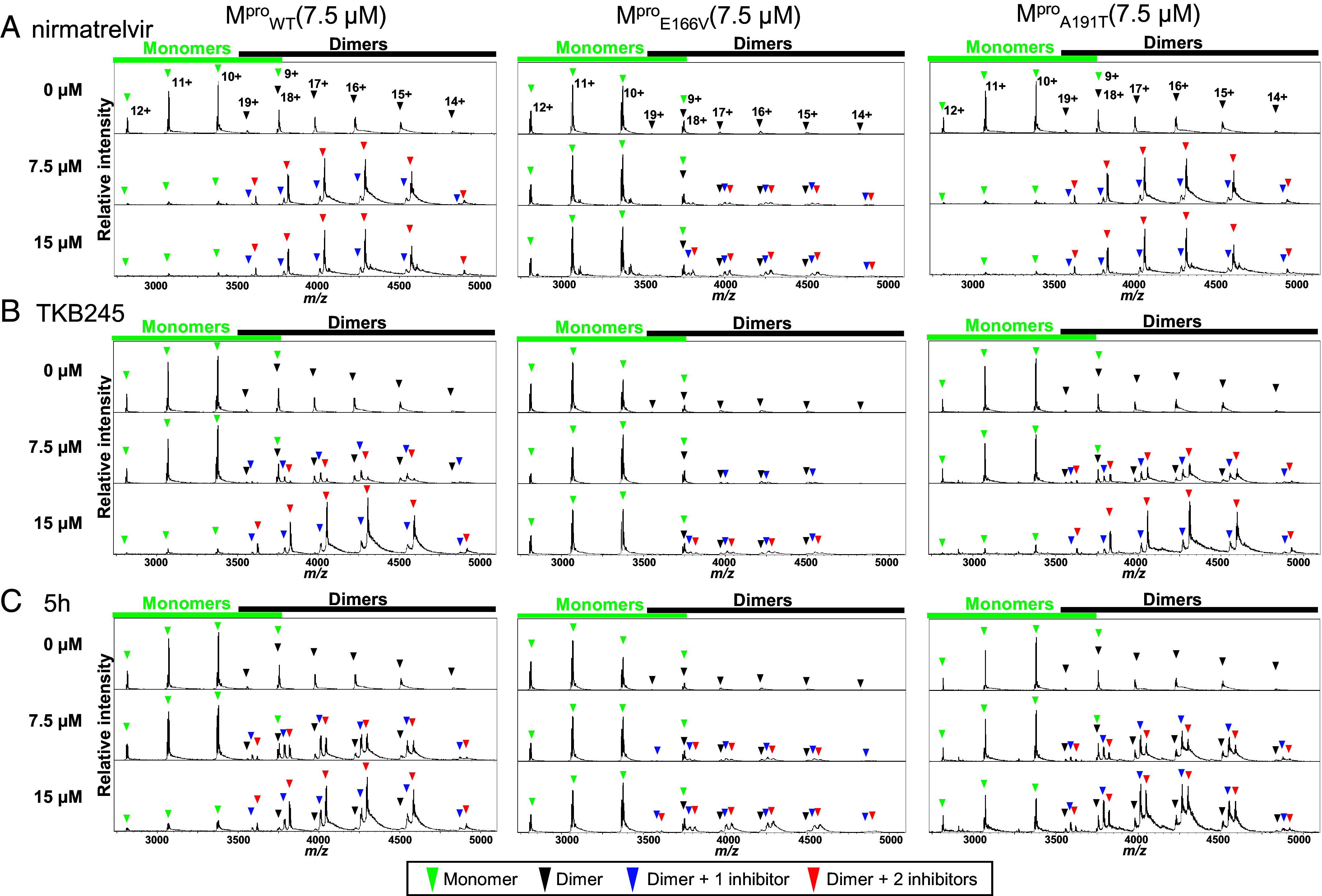
M^P^I binds to M^pro^ and promotes its dimerization. M^pro^_WT_, M^pro^_E166V_, or M^pro^_A191T_ (all at 7.5 µM) was exposed to 0, 7.5, or 15 µM of each M^P^I in 10 mM ammonium acetate and subjected to native mass spectrometric analysis. Relative native mass spectra with nirmatrelvir (*A*), TKB245 (*B*), or 5h (*C*) are shown. Charge states 9^+^, 10^+^, 11^+^, and 12^+^ are annotated to mass spectra corresponding to monomeric M^pro^ species and charge states 14^+^, 15^+^, 16^+^, 17^+^, 18^+^, and 19^+^ are annotated to mass spectra corresponding to dimeric M^pro^ species. The peaks are annotated with green reversed triangles for monomers, black for dimers, blue for dimers bound by one inhibitor molecule, and red for dimers bound by two inhibitor molecules.

We examined the modes of the binding of TKB245 to M^pro^_WT_ and M^pro^_E166V_ using native mass spectrometry (Native MS) analysis. In the absence of inhibitors, the monomer-dimer equilibrium of M^pro^_WT_ (7.5 µM) shifted toward more abundant monomers with fewer dimers (Fig. 3 *A*, *Left* *Top*), but in the presence of 7.5 and 15 µM nirmatrelvir, the equilibrium remarkably shifted to dimers with at most two nirmatrelvir molecules bound (Fig. 3 *A*, *Left* *Middle* and *Bottom*, respectively). M^pro^_E166V_ was also found shifted toward more abundant monomers in the absence of inhibitors (Fig. 3 *A*, *Middle* *Top*), but there was no shift to dimers even in the presence of 15 µM nirmatrelvir (Fig. 3 *A*, *Middle* *Bottom*), suggesting that, with E166V substitution, dimerization process failed to occur, consistent with the structural data showing the disruption of Ser1’-Glu166 interactions (Fig. 2 *B*, *c* and *d*). In the presence of 7.5 or 15 µM TKB245, there was also a remarkable concentration-dependent shift to the dimeric form with at most two TKB245 molecules bound (Fig. 3 *B*, *Left* *Bottom*). However, with M^pro^_E166V_ there was essentially no remarkable shift to dimers (Fig. 3 *B*, *Middle*) as in the case of nirmatrelvir (Fig. 3 *A*, *Middle*). These data together strongly suggest that the dimerization of M^pro^_E166V_ protomers was hindered by the E166V substitution due to the disruption of the presumed dimerization-initiating Ser1’-Glu166 interactions.

When M^pro^_A191T_ was examined in association with nirmatrelvir (7.5 and 15 µM; Fig. 3 *A*, *Right*) and TKB245 (7.5 and 15 µM; Fig. 3 *B*, *Right*) in further native mass spectrometric analysis, dimerization of M^pro^_A191T_ protomers clearly occurred, and the dimerization profile of those protomers was virtually the same as seen with the dimerization of M^pro^_WT_ protomers (*Left* panels in Fig. 3 *A* and *B*), suggesting that the A191T substitution exerts no significant effects to the dimerization process. Indeed, the region around Ala191 does not participate in the dimerization process, as the dimer interface is situated more distantly, primarily within the helical domain. X-ray crystallographic data also showed that the interactions of Ser1’ and Glu166 of M^pro^_A191T_ complexed with 5h (*SI Appendix*, Fig. S4) were virtually the same compared to those of M^pro^_WT_ complexed with nirmatrelvir or TKB245 (Fig. 2*B*).

The A191T substitution occurred when SCoV2^WK521^_WT_ was propagated in the presence of 5h and the virus acquired substantial resistance to 5h (Fig. 1*D* and *SI Appendix*, Table S1). Thus, we also examined the mode of 5h binding to M^pro^_WT_ and M^pro^_A191T_. As we previously reported (20), based on a crystal structure, 5h does form a covalent bond with M^pro^_WT_ as observed in the X-ray crystallographic analyses. Indeed, 5h (15 µM) elicited the dimerization of M^pro^_WT_ (Fig. 3 *C*, *Left*) comparably as TKB245 did (Fig. 3 *B*, *Left*). However, when M^pro^_E166V_ was examined with 5h, no remarkable shift was observed (Fig. 3 *C*, *Middle*), suggesting that the E166V substitution also substantially blocked the binding of 5h to M^pro^_E166V_. Interestingly, 5h presumably bound to M^pro^_A191T_ but with predominantly one 5h molecule bound and elicited its dimerization to less extent compared to the cases of nirmatrelvir and TKB245 examined with M^pro^_A191T_ (Fig. 3 *C*, *Right*). The less-binding profile of 5h to M^pro^_A191T_ is in line with the structural features showing that A191T substitution disrupts the interactions between the distal ring of the indole moiety of 5h and Ala191, pushing 5h molecule away from M^pro^_A191T_ (Fig. 2 *C*, *b*–*d*), resulting in less anti-viral potency of 5h with M^pro^_A191T_. Such disruption does not occur as nirmatrelvir (Fig. 2 *C*, *e*).

### Replicability of SCoV2^WK521^_WT_, SCoV2^WK521^_E166V_, and SCoV2 ^WK521^_A191T_ as Examined in CSRA.

**Fig. 4.**
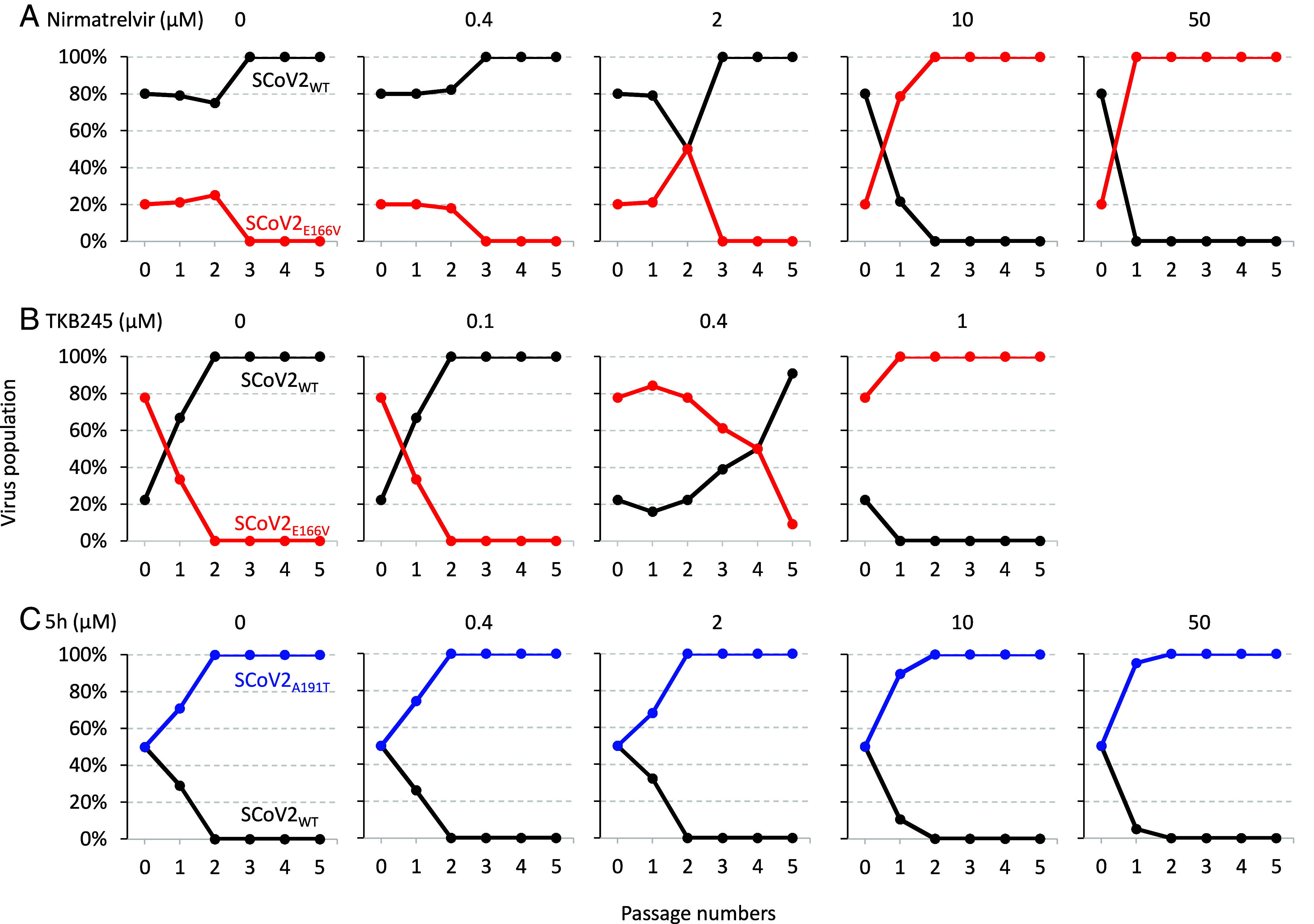
CSRA for M^P^I-resistant SCoV2. Replication profiles of SCoV2^WK521^_E166V_ and SCoV2^WK521^_A191T_ were examined in CSRA. Each SCoV2 variant contained dominantly selected mutations in M^pro^, E166V, and A191T in the presence of nirmatrelvir (*A*) or TKB245 (*B*) and 5h (*C*), respectively. Two SCoV2 variants to be compared for their fitness were mixed 50:50 and used to infect VeroE6^TMPRSS2^ cells in the presence of various concentrations of nirmatrelvir or 5h. The cell-free supernatant was transferred to fresh VeroE6^TMPRSS2^ cells every 3 to 5 d. Viral RNA extracted from supernatants at the end of each passage was subjected to Sanger sequencing, and the proportions of Glu and Val at position 166 in M^pro^ and those of Ala and Thr at position 191 were determined.

We finally examined whether SCoV2 fitness was altered upon the acquisition of E166V or A191T substitution in M^pro^, which apparently confers on SCoV2 resistance to nirmatrelvir, TKB245, and/or 5h, using a CSRA in a setting of the SCoV2^WK521^_WT_ genetic background. Target VeroE6^TMPRSS2^ cells were exposed to a mixture of paired infectious viral populations (SCoV2^WK521^_WT_ + SCoV2^WK521^_E166V_ and SCoV2^WK521^_WT_ + SCoV2^WK521^_A191T_), and SCoV2 in the culture supernatant was transmitted to new cultures every 3 to 5 d. The M^pro^-encoding region of the virus was Sanger-sequenced at various time points, and the relative proportion of the two viral populations was determined (*SI Appendix*, Fig. S6). When the assay was begun in the absence of nirmatrelvir using an 80:20 mixture of SCoV2^WK521^ and SCoV2^WK521^_E166V_ viral populations, the percentage of SCoV2_E166V_ rapidly decreased to virtually 0% by passage 3 (Fig. 4 *A*, the very *Left* panel). On the other hand, the percentage of SCoV2^WK521^, initiated with ~80%, increased to 100% by passage 3 (Fig. 4 *A*, the very *Left* panel). In the presence of two relatively low concentrations of nirmatrelvir (0.4 and 2 µM), the replication pattern did not change drastically. However, when the assay was conducted in the presence of 10 µM nirmatrelvir, the population of SCoV2^WK521^_E166V_ dominated SCoV2^WK521^ by passage 2, and the percentage of SCoV2^WK521^_E166V_ reached ~100% by passage 2 (Fig. 4 *A*, the second-from-very *Right* panel). In the presence of 50 µM nirmatrelvir, the percentage of SCoV2^WK521^_E166V_ reached ~100% by passage 1 (Fig. 4 *A* the very *Right* panel). These data strongly suggest that in the absence of nirmatrelvir, the fitness of SCoV2^WK521^_E166V_ was inferior to that of SCoV2^WK521^ and that in the presence of >10 µM nirmatrelvir, SCoV2^WK521^_E166V_ predominates SCoV2^WK521^ rapidly or virtually immediately during the passages (Fig. 4*A*). In addition, as shown in Fig. 4*B*, CSRA demonstrated in the presence of low concentrations of TKB245 (0.1 and 0.4 µM), the replicability of SCoV2^WK521^_WT_ was greater than that of SCoV2^WK521^_E166V_ and the SCoV2^WK521^_WT_ population reached 100% by passage 5, while in the presence of a higher concentration of TKB245 (1 µM), the replicability of SCoV2^WK521^_WT_ was less and the SCoV2^WK521^_E166V_ population reached 100% by passage 1.

We also asked whether the replication fitness of SCoV2^WK521^_A191T_ was greater than that of SCoV2^WK521^ and conducted the CSRA using a 50:50 mixture of SCoV2^WK521^ and SCoV2^WK521^_A191T_ viral populations. In the absence of 5h, the percentage of SCoV2^WK521^_A191T_ became ~100% and that of SCoV2^WK521^ went down to ~0% by passage 2, indicating that SCoV2^WK521^_A191T_ had increased its replicability surpassing SCoV2^WK521^’s replicability. The EC_50_ of SCoV2^WK521^_A191T_ against 5h was >100 µM (*SI Appendix*, Table S1), and as expected, in the presence of wide concentrations of 5h (0.4 to 50 µM), the pattern of replicability remained the same and SCoV2^WK521^_A191T_ predominated SCoV2^WK521^ throughout all the concentrations tested (Fig. 4*C*). These data, considering that while A191T substitution confers 5h-resistance on SCoV2, there are no other structural changes resulting in increased replicability, A191T substitution is likely to be associated with increased viral fitness to SCoV2^WK521^_A191T_. The functional mechanism for the increased viral fitness remains to be elucidated.

We also asked whether the L50F substitution, which has reportedly been associated with the viral replication fitness (12, 17), affected the replication fitness of rgSCoV2_E166V_ using the CSRA (26, 27). As shown in new S*I Appendix*, Fig. S7, by passage 1, the rgSCoV2_L50F/E166V_ population reached 100% and that of rgSCoV2_E166V_ dropped down to 0%, indicating that the replicability of rgSCoV2_L50F/E166V_ substantially surpassed that of rgSCoV2_E166V_. These data strongly suggest that the L50F substitution compensates the loss of replicating fitness caused by the E166V substitution.

## Discussion

In the present study, when the ancestral SCoV2^WK521^ was passaged in the target VeroE6^TMPRSS2^ cells every 3 d in the presence of increasing concentrations of each M^P^I, only a couple of amino acid substitutions were identified (Fig. 1). The substitution patterns identified include i) E166V or E166V+L50F when selected with nirmatrelvir; ii) L50F or E166V+L50F when selected with TKB245; and iii) A191T alone when selected with 5h. By contrast, when Iketani and his group passaged an ancestral SCoV2 strain 16 times in the presence of increasing concentrations of nirmatrelvir in ACE2-expressing Huh7 cells, 53 independent viral lineages were seen and resultant substitutions were identified in 23 different residues in the M^pro^ (12). In their study, the E166V mutation conferred the strongest resistance (around 100-fold), but this E166V substitution apparently resulted in a loss of viral replicative fitness that was restored by compensatory changes such as L50F and T21I. Heilmann et al. also passaged an ancestral SCoV2 strain in the baby hamster kidney-21 (BHK-21) cells 3 times, and found 39 distinct amino acid substitutions distributed over the entire sequence of M^pro^, including F3S, Y54C/S, G138S, L141F, L167F, Q192R, D197Y, R298G, Q299K/P, and F305L (28). These data suggest that while a variety of amino acid substitutions reportedly emerge in M^pro^, the E166V substitution is at least one of the most important substitutions in M^pro^ when exposed to nirmatrelvir. One of the reasons why only E166V or E166V+L50F substitutions emerged in our study could be that since the susceptibility of VeroE6^TMPRSS2^ cells to the infectivity of the virus is very high. This nature of the VeroE6^TMPRSS2^ cells possibly made the “drug pressure” given from the continuous presence of nirmatrelvir much greater than the use of other cells previously reported so that only a few and the most important nirmatrelvir-resistance-associated amino acid substitutions (E166V and L50F) emerged in the present study.

As such, the present study identified E166V in M^pro^ as a nirmatrelvir-associated substitution and revealed that M^pro^_E166V_ causes the loss of structural interactions with nirmatrelvir (Fig. 2*B*), preventing the dimerization of M^pro^_E166V_ (Fig. 3), which resulted in the acquisition SCoV2^WK521^_E166V_ of high-level resistance to nirmatrelvir. Of note, in the presence of high concentrations (10 and 50 µM) of nirmatrelvir, the nirmatrelvir -associated variant SoV2^WK521^_E166V_ predominant SCoV2^WK521^_WT_. In these replicability results (Fig. 4) strongly suggest that this SCoV2 dominance may occur in those infected with SCoV2 receiving nirmatrelvir and the risk of its transmission to other individuals is possible, although such issues remain to be further study.

Regarding the possible nature of the L50F substitution, two research groups have previously investigated the structural profile of M^pro^_L50F_ (16, 29). Duan et al. elucidated the mechanism by highlighting increased hydrophobic interactions between F50 and Q189, resulting in a conformational change in the hydrophobic S2 pocket, whereas Chen et al. suggested the involvement of additional residues, particularly M49 and R188, based on structural deviations. Our observations illustrated in *SI Appendix*, Fig. S3 indicate direct involvement of both Q198 and M49 in the binding pocket, likely influencing substrate binding, while R188, although exhibiting structural movement, is situated further away from the binding pocket. This movement may arise from the inherent flexibility of the arginine side chain itself rather than from the L50F substitution. Nevertheless, our data from the CSRA indicate that the L50F substitution compensates for the replicability of SCoV2 supports previous reports (12, 17). In this regard, it is likely that the CSRA is more sensitive in detecting functional differences with or without the L50F substitution compared to the structural differences identified through rather static X-ray crystallography. Concerning nirmatrelvir-resistance-associated amino acid substitutions, it is noteworthy that Lewandowski and his group have shown that the binding of M^pro^ substrate may involve more than residues in the active site: in particular, L50F and other nonactive site amino acid substitution may enhance the M^pro^ dimer–dimer interactions and help place the nsp5-6 substrate at the enzyme catalytic center (30).

In the selection with 5h, the virus well replicated even in the presence of over 100 µM of 5h (Fig. 1*D*). The finding that A191T substitution was identified as quickly as by passage 5 (Fig. 1 and *SI Appendix*, Fig. S1*C*) may suggest that the SCoV2 might have contained the A191T substitution-carrying viruses as polymorphism at the outset. Thus, under the selection with 5h, the A191T substitution-carrying viral population was selected and became dominant.

In the present study, in an attempt to analyze how M^P^I-resistant SCoV2 variants would emerge during propagation under continuous exposure to M^P^I, we employed a clinical SCoV2 isolate (the ancestral SCoV2^WK521^), but not a clonal viral strain generated by a genetic engineering method such as reverse genetic techniques (22, 31). SCoV2^WK521^_WT_ was originally isolated from an individual infected by an SCoV2 strain and had been propagated and passaged in VeroE6^TMPRSS2^ cells from the initial culture and should have contained a variety of amino acid substitutions compared to its original genetic sequence. Some of such amino acid substitutions would serve as naturally occurring polymorphisms, which would result under inhibitory pressures in infected individuals including immunologic and innate antiviral factors. Indeed, in the case of the selection of anti-HIV-1-drug-resistant variants, highly drug-resistant variants more rapidly emerge under drug selection pressure when propagated with the mixture of multiple drug-resistant clinical strains than recombinant infectious clones (32). Thus, we also examined the drug-resistance profiles of the variants by using the viral populations following the selection, while we also employed reverse genetic technique-yielded recombinant SCoV2 strains containing the targeted amino acid substitution(s) for confirmatory experiments (*SI Appendix*, Table S3).

The X-ray crystallography data with mass spectrometry data obtained in the present study clearly show that all three M^pro^ inhibitors specifically bind to the enzymatic active site of the enzyme through the formation of covalent, polar, (and halogen) binding interactions, causing the dimerization of two enzyme protomers. Each inhibitor blocks the entry of the premature viral polyproteins into the active site cavity, thus presumably blocking the enzymatic processing and maturation of the viral polyproteins. If the affinity of M^pro^ inhibitors to the enzyme is high, the inhibitors would strongly stay bound, thus blocking the entry of the polyproteins and inhibiting the enzymatic processing. The absence of the complex of nirmatrelvir with M^pro^_E166V_ in the present X-ray crystallographic data strongly suggests that the affinity of nirmatrelvir to M^pro^_E166V_ is not good enough to form the complex, differing from the feature of TKB245 with M^pro^_E166V_, at least under the conditions of our attempt to make crystals. Thus, the data obtained from the dimerization profiles and mass spectrometry are highly relevant to physiology of interactions between M^pro^ and inhibitors.

In conclusion, the present data show that the structural changes in the target enzyme (e.g., M^pro^ in the current work), which certain amino acid substitutions bring about, compromise the enzyme’s integrity (e.g., protomer dimerization and the following structural changes), loosen the binding of potential therapeutics to the enzyme and cause resistance. However, certain structural modifications and optimizations of the potential therapeutics tighten the loosened interactions and restore their antiviral activity. The data in this work also shed light in the understanding of the mechanisms of the emergence of resistant viruses and the development of antiviral agents which does not cause resistance in the pathogens and remain active against them.

## Materials and Methods

### Cells, Viruses, and Antiviral Compounds.

TMPRSS2-expressing VeroE6 (VeroE6^TMPRSS2^) cells (33) and hACE2- and TMPRSS2-expressing Hela (Hela^hACE2/TMPRSS2^) cells were obtained from Japanese Collection of Research Bioresources Cell Bank (JCRB1819 and JCRB1835, respectively). Both cells were maintained in DMEM supplemented with 10% FCS, penicillin (100 µg/mL), kanamycin (50 µg/mL), and G418 (0.5 mg/mL). SARS-CoV-2 strain 2019-nCoV/Japan/TY/WK-521/2020 (EPI_ISL_408667, Wuhan ancestral) was obtained as a wild-type strain (SCoV2^WK521^_WT_) from National Institute of Infectious Diseases (Tokyo, Japan), propagated in highly SCoV2-susceptible VeroE6^TMPRSS2^ cells (33), and its authenticity was validated with deep sequencing before its use for selection assays. Nirmatrelvir was purchased from Med Chem Express (#HY-138687) (Monmouth Junction, NJ, USA). TKB245 (18, 20) and 5h (19) were synthesized by H. Tamamura (Tokyo Medical and Dental University, Tokyo, Japan) and by A. K. Ghosh (Purdue University, West Lafayette, IN), respectively. Each compound was dissolved in DMSO at 20 mM and served as a stock solution.

### In Vitro Selection of Drug-Resistant Variants.

We attempted to select SCoV2 variants resistant to nirmatrelvir, TKB245, and 5h by propagating SCoV2^WK521^ in VeroE6^TMPRSS2^ cells (10^5^ cells/well in a 24-well plate) in the presence of increasing concentrations of each antiviral agent. When the replication of the virus was confirmed as the viral cytopathic effect (CPE) under microscopy in 3 to 5 d from the start of selection, the supernatants were harvested. The next round of selection was started in the presence of 0.5-, 1-, and 2-fold greater concentrations of each agent. If sufficient levels of CPE were observed, the highest concentration of the agent was used for the following passage, and the culture supernatants were stored at −80 °C in each passage.

### Sequencing.

To determine the nucleotide and amino acid sequences, cell-free RNA was extracted and subjected to Sanger sequencing or deep sequencing. The sequencing was performed by Eurofins Genomics (Ebersberg, Germany) or SB novel coronavirus test center (Chiba, Japan). In brief, Sanger sequencing was conducted using the following primers: F1 (5′- CTT GTT GTC ATC TCG CAA AG -3′), F2 (5′- TTG TTG ACA GGC AAA CAG C -3′), R1 (5′- TCG ATT GAG AAA CCA CCT GT -3′), and R2 (5′- ACC ATC ATC ATA CAC AGT TCT -3′). Deep sequencing analyses were performed using RNA extracted from culture supernatants obtained at appropriate passages. RNA extracted from the nirmatrelvir-10-passage SCoV2^WK521^ (SCoV2^WK521^_NIR-P10_), TKB245-9-passage SCoV2^WK521^ (SCoV2^WK521^_TKB245-P9_), TK245-15-passage SCoV2^WK521^ (SCoV2^WK521^_TKB245-P15_), and 5h-9-passage SCoV2^WK521^ (SCoV2^WK521^_5h-P9_) were subjected to deep sequencing using Illumina COVIDseq with the ARTIC V4.1 protocol and iSeq100 or NextSeq 2000. The data obtained were assembled using BaseSpace DRAGEN COVID Lineage v3.5.12. To get the percentages of the virus with specific amino acid substitutions, mutation analysis was performed using Mutations Analysis Program (Coronavirus Antiviral & Resistance Database, Stanford University, CA) (https://covdb.stanford.edu/sierra/sars2/by-patterns/). The accession numbers obtained from NCBI-SRA are as follows: SRR27685484 (for SCoV2^WK521^_NIR-P10_), SRR27688145 (SCoV2^WK521^_TKB245-P9_), SRR27688221 (SCoV2^WK521^_TKB245-P15_), and SRR27685483 (SCoV2^WK521^_5h-P9_). In SCoV2^WK521^_NIR-P10_ and SCoV2^WK521^_5h-P9_, two dominant amino acid substitutions, E166V and A191T, were identified and designated in the present work as SCoV2^WK521^_E166V_ and SCoV2^WK521^_A191T_, respectively.

### Antiviral Assays.

For antiviral assays, VeroE6^TMPRSS2^ cells or Hela^hACE2/TMPRSS2^ cells were seeded in 96-well microtiter culture plates and incubated overnight. Next day, the virus was inoculated onto the cells at MOI = 0.5, cultured for 1 h, the cells were washed to remove the excess viruses, and various concentrations of antiviral compounds were added into the wells. After an additional 2 to 3 d, cell culture supernatants were harvested, and viral RNA extracted and subjected to quantitative RNA-PCR (RNA-qPCR) using the following primers and a probe: 5′-AAA TTT TGG GGA CCA GGA AC-3′, 5′-TGG CAG CTG TGT AGG TCA AC-3′, and 5′-FAM-ATG TCG CGC ATT GGC ATG GA-black hole quencher 1 (BHQ1)-3′.

### Protein Expression and Purification.

The SCoV2-M^pro^-encoding genes were cloned into pGEX-4T1 vector (Genscript) with N-terminal self-cleavage site (SAVLQ/SGFRK). At the C terminus; the construct codes for the HRV 3 C PreScission protease cleavage site (SGVTFQ ↓ GP) connected to a 6His tag. E166V and A191T mutations were introduced using the Q5 Site-Directed Mutagenesis Kit (NEB, Ipswich, MA, USA). The plasmid constructs were transformed into BL21 Star (DE3) cells (Thermo Fisher Scientific). The cultures were grown in LB media supplemented with 50 µg/mL of ampicillin. Protein expression was induced by adding 1 mM IPTG at an optical density of 0.6 to 0.8 at 600 nm and the cultures were maintained at 20 °C overnight. SCoV2-M^pro^ preparations were purified first by affinity chromatography using Ni-NTA agarose (FUJIFILM Wako Pure Chemical Corp., Osaka, Japan). The authentic N terminus is generated by M^pro^ autoprocessing during expression, whereas the authentic C terminus is generated by the treatment with PreScission (Cytiva, Tokyo, Japan) protease. The resulting authentic 306 amino acid M^pro^ was further purified by SEC using a HiLoad Superdex 200 pg column (Cytiva) in 20 mM Tris, pH 7.5, 150 mM NaCl, and 1 mM DTT.

### Biochemical Assays of M^pro^.

The profiles of M^pro^ were determined by measuring changes in fluorescence resonance energy transfer (FRET) on 14-mer fluorogenic peptide substrate, DABCYL-KTSAVLQSGFRKME-EDNAS (BPS Bioscience, San Diego, CA). Measurements were performed with the substrate (20 μM) in 3CL Protease Assay Buffer (BPS Bioscience) in the presence or absence of each M^P^I. Activity was measured every 2 min for up to 60 min at 25 °C on a Cytation 5 cell imaging multimode reader (BioTek, Winoosk, VT, USA) using a monochromator (Ex: λ = 360 nm/Em: λ = 460 nm). The Ki, K_m_, and k_cat_ values were determined using GraphPad Prism 9.5.1 (GraphPad, San Diego, CA).

### Reverse Genetics to Produce Recombinant SCoV2 from a Bacterial Artificial Chromosome (BAC) Encoding the Viral Full-Genome Sequence.

The full-genome nucleotide sequence of SCoV2 (Wuhan/Hu-1/2019, NC_045512) with or without the E166V or L50F/E166V substitution in Nsp5 was assembled into the pBeloBAC11 vector to generate infectious cDNA clones under the control of a cytomegalovirus promoter by using Gibson Assembly Master Mix (NEB) as described previously (22, 23). To rescue the recombinant SCoV2, the BACs were transfected into HEK293T cells. Three days post-transfection, the supernatant was inoculated onto VeroE6^TMPRSS2^ cells to prepare virus stocks. The virus titers of the stock viruses were determined by using plaque assays using VeroE6^TMPRSS2^ cells. The stock reverse-genetic-generated viruses (rgSCoV2s) were subjected to deep sequencing as described elsewhere (34) to confirm the absence of unwanted mutations. All experiments with the rgSCoV2 preparations were performed in enhanced biosafety level 3 (BSL3) containment laboratories at the University of Tokyo, complying with the rules and regulations regarding the safety and ethics (approved ID:5-517) set by the Japanese Government throughout the present study.

### X-ray Structural Analyses of Nirmatrelvir, TKB245, or 5h Complexed with M^pro^ Species.

The recombinant M^pro^ preparations (M^pro^_WT,_ M^pro^_E166V_, and M^pro^_A191T_) were concentrated up to 7.1 mg/mL and incubated with 600 μM nirmatrelvir, TKB245, or 5h for 1 h before crystallization. Crystals were grown using the sitting drop vapor diffusion method at 20 °C. The reservoir solution contained 28% v/v 2-propanol, 0.1 M Bis-Tris pH 6.5, 3% v/v PEG-200 for M^pro^_WT_ in complex with TKB245, 0.1 M HEPES pH 7.5, 16% w/v PEG-3350 for M^pro^_E166V_-TKB245 complex and 3% DMSO or 0.1 M MES pH 6.8, 15% PEG-6000 for apo M^pro^_E166V_. Crystals were soaked briefly in a cryoprotection solution containing 20% glyserol. X-ray data for TKB245 and 5h inhibitors were collected at the National Synchrotron Light Source II BEAMLINE 19-ID (Upton, NY, USA), while X-ray data for M^pro^ attempted to complex with nirmatrelvir were collected at SPring-8 BL41XU (Hyogo, Japan) and processed using DIALS (integrated within CCP4i2) via xia2 (35, 36). Data collection statistics are shown in *SI Appendix*, Table S4. The phase problem was solved by molecular replacement using MolRep (37) using the previously deposited M^pro^ structures (PDB IDs: 8DOX, 8UH9) as models. All water molecules and ligand atoms were omitted from the starting model. Subsequent cycles of refinement were performed in REFMAC5 (38). Structure files of nirmatrelvir, TKB245, and 5h were manually fitted to the electron density. All structural figures were produced with the PyMOL Molecular Graphics System (Schrödinger, LLC). The crystallographic data of M^pro^_WT_ in complex with TKB245, M^pro^_E166V_ in complex with TKB245, M^pro^_E166V_ failing to complex with nirmatrelvir, and M^pro^_A191T_ in complex with 5h were deposited to PDB and their ID obtained are 9ARQ, 9ARS, 9ART, and 8UH8, respectively.

### Native MS.

The purified SCoV2-M^pro^ (with or without E166V or A191T substitution) preparations were buffer-changed using PD-10 desalting columns (Cytiva) equilibrated in 10 mM ammonium acetate (pH 6.7). Each SCoV2-M^pro^ preparation (7.5 µM) was exposed to respective M^P^I of final concentrations of 0, 7.5, or 15 µM. Native MS was conducted using the ESI-QTOF mass spectrometer (Impact II, Bruker Daltonics, Bremen, Germany) with the LC system (Prominence UFLC, Shimadzu, Kyoto, Japan). Injection volume and flow rate of sample introduction were 10 µL and 0.5 mL/min, respectively. Samples were ionized in positive ion mode with the following ion source parameters: dry heater: 200 °C, nebulizer: 3.0 bar, dry gas: 10.0 L/min, capillary voltage: 4,500 V, end plate offset: −400 V, charging voltage: 2,000 V. MS scans have been acquired at a spectra rate of 1 Hz at a mass range from 100 to 6,000 m/z. Molecular masses were determined by protein deconvolution using DataAnalysis 4.4 (Bruker Daltonics). All species detected by native MS including their theoretical molecular masses, experimental molecular masses, and mass errors are listed in *SI Appendix*, Fig. S5.

### CSRA.

Two titrated infectious viral populations to be examined in the CSRA, which best approximated 20:80 and 50:50 mixtures were inoculated onto freshly prepared VeroE6^TMPRSS2^ (2 × 10^5^/mL) in the presence or absence of various concentrations of nirmatrelvir or 5h. After 3 to 5 d of coculture, the supernatants of the coculture were harvested and the supernatants were transferred (1/100 volume) onto newly prepared uninfected freshly prepared VeroE6^TMPRSS2^ cells (2 × 10^5^/mL) for the next round of the passage. At the end of each passage, viral RNA extracted from the supernatants was subjected to Sanger sequencing mentioned above, and the emergence of amino acid substitutions was examined.

## Supplementary Material

Appendix 01 (PDF)

## Data Availability

The X-ray crystallographic data of M^pro^_WT_ in complex with TKB245, M^pro^_E166V_ in complex with TKB245, M^pro^_E166V_ failing to complex with nirmatrelvir, and M^pro^_A191T_ in complex with 5h were deposited in PDB and their ID obtained are 9ARQ, 9ARS, 9ART and 8UH8, respectively. All deep sequencing data in the fastq file format are deposited in the Sequence Read Archive under BioProject accession no. PRJNA10496411 (39). All other study data are included in the article and/or *SI Appendix*.
