# Supplementary material for: Structural and virologic mechanism of the emergence of resistance to Mpro inhibitors in SARS-CoV-2
Source: Proc Natl Acad Sci U S A. 2024 Sep 5;121(37):e2404175121. doi: 10.1073/pnas.2404175121 (PMC11406233; doi:10.1073/pnas.2404175121)
Supplement: Supplementary file 1 — Appendix 01 (PDF) [file pnas.2404175121.sapp.pdf]

PNAS

Supporting Information for

Structural and virologic mechanism of the emergence of resistance to M^{pro} inhibitors in SARS-CoV-2

Shin-ichiro Hattori *et al.*

This PDF file includes:

Figures S1 to S7
Tables S1 and S4

Supporting Figures and Tables

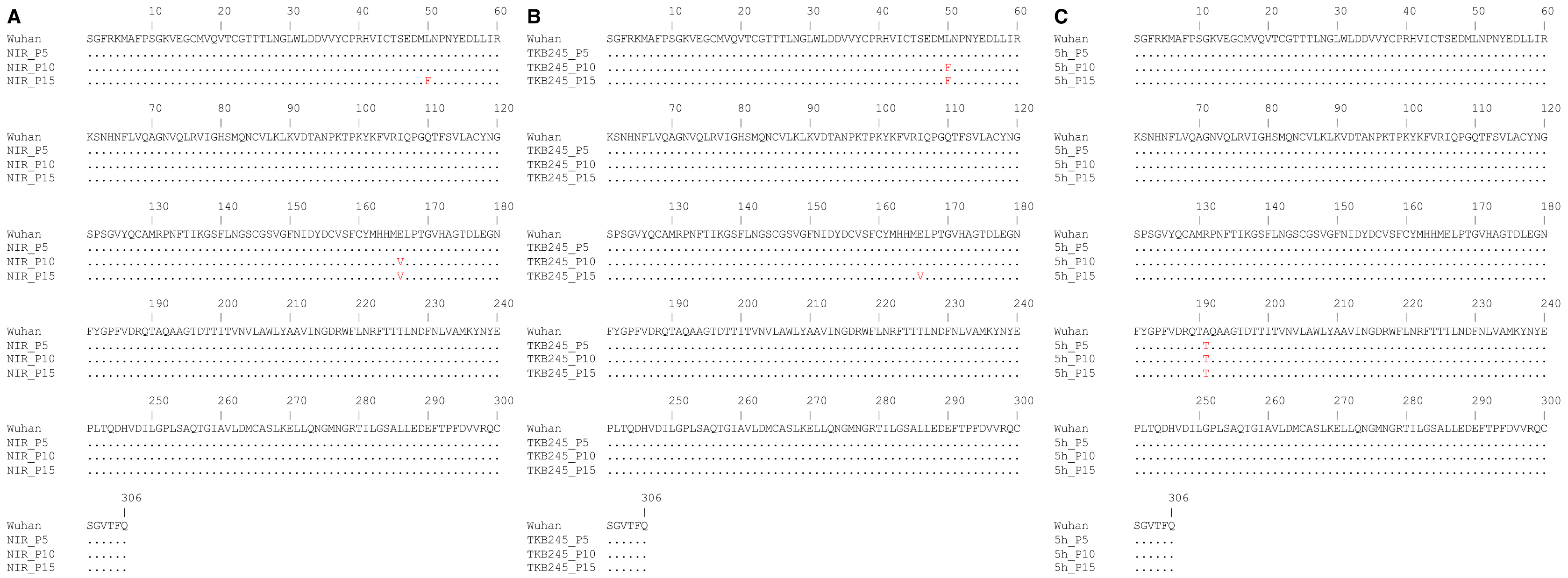

Fig. S1. Amino acid substitutions emerged in SCoV2's M^{pro} under the drug pressure of test compounds. Amino acid changes in M^{pro} at passages 5, 10, and 15 in the presence of nirmatrelvir (**A**), TKB245 (**B**), and 5h (**C**), respectively, were determined with Sanger sequencing method. The substitutions identified are highlighted in red.

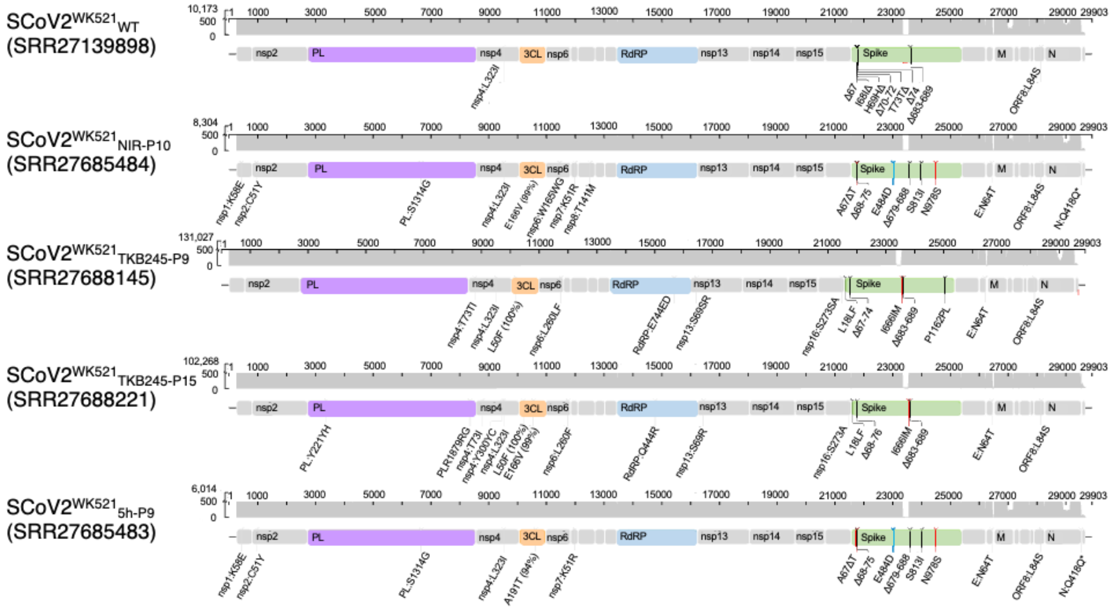

Fig. S2. Amino acid substitutions in the entire genome of SCoV2 selected with test compounds. RNA extracted from $SCoV2^{WK521}_{WT}$, nirmatrelvir-10-passaged SCoV2 ($SCoV2^{WK521}_{NIR-P10}$), TKB245-9-passaged SCoV2 ($SCoV2^{WK521}_{TKB245-P9}$), TKB245-15-passaged SCoV2 ($SCoV2^{WK521}_{TKB245-P15}$), and 5h-9-passaged SCoV2 ($SCoV2^{WK521}_{5h-P9}$) was subjected to next-generation sequencing using Illumina COVIDseq with the ARTIC V4.1 protocol and iSeq100. The data obtained were assembled using BaseSpace DRAGEN COVID Lineage v3.5.12. Mutation analysis was performed using Mutations Analysis Program (Stanford University, Coronavirus Antiviral & Resistance Database, https://covdb.stanford.edu/sierra/sars2/by-patterns/). Amino acid substitutions identified are illustrated using Mutations Analysis Program (https://covdb.stanford.edu/sierra/sars2/by-patterns/). The sequences are aligned to the reference sequence $SCov2^{Wuhan-Hu-1}$ (NC_045512.2).

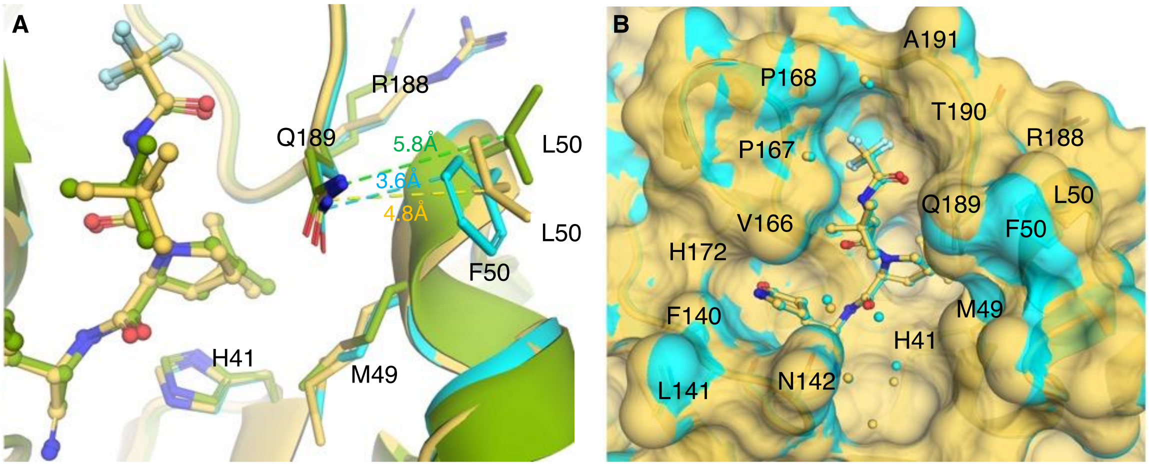

Fig. S3. L50F substitution in M^{pro} does not affect the size or shape of M^{pro}'s binding pocket for nirmatrelvir. (A) Superimposition of crystal structures of M$^{pro}_{WT}$ (green), M$^{pro}_{E166V}$ (yellow), and M^{pro} with two substitutions (M$^{pro}_{L50F/E166V}$ in cyan) in complex with nirmatrelvir (cyan). Note that when E166V is present, nirmatrelvir is not complexed with M$^{pro}_{E166V}$. Nirmatrelvir is illustrated with surrounding residues in stick form. The active site residues occupy nearly identical positions with minimal deviations among the three structures. The PDB IDs are: M$^{pro}_{WT}$ (7VH8), M$^{pro}_{E166V}$ (8H82), and M$^{pro}_{L50F/E166V}$ (8H5P). (B) Superimposition of the three crystal surfaces: M$^{pro}_{E166V}$ (in yellow) and M$^{pro}_{L50F/E166V}$ (cyan) in complex with nirmatrelvir. The surface representation of the binding pocket also exhibits virtually identical features. The L50F substitution, located adjacent to the binding pocket, predominantly interacts closely with Q189 but does not induce any discernible alterations in its position. The PDB IDs are 8H82 for M$^{pro}_{E166V}$ and 8H5P for M$^{pro}_{L50F/E166V}$.

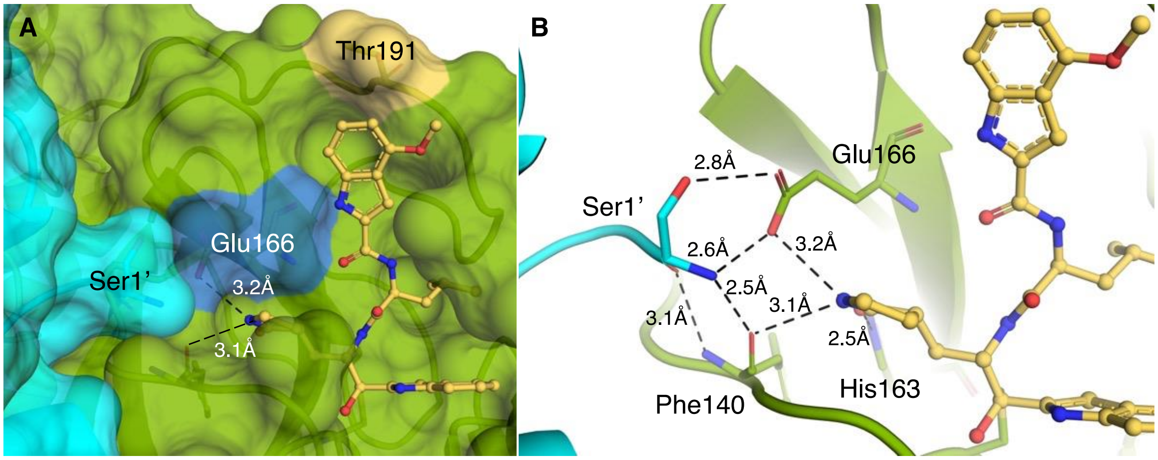

Fig. S4. A191T substitution does not affect the Ser1' and Glu166 interactions critical for M^{pro} protomer dimerization. (A) X-ray crystal structure of M$^{pro}_{A191T}$ complexed with 5h (yellow sticks) featuring the dimer interface of the enzyme. Note that A191T substitution does not affect the Ser1'-Glu166 interactions. (B) The tight hydrogen bond interaction network (black dashed lines) formed is associated with 5h. The location of Thr191 is distant from the network as shown in (A) and is not seen in this figure.

Species of M^{pro}_{WT}	Theoretical mass	Experimental mass*	Mass error	Fig. 3
M^{pro} (monomer)	33796.6	33795.7	-0.9	A
2x M^{pro} (dimer)	67593.3	67591.4	-1.9	A
2x M^{pro} + 1x Nirmatrelvir	68092.8	68089.9	-2.9	A
2x M^{pro} + 2x Nirmatrelvir	68592.3	68591.6	-0.7	A

Species of M^{pro}_{WT}	Theoretical mass	Experimental mass*	Mass error	Fig. 3
M^{pro} (monomer)	33796.6	33795.6	-1.0	B, C
2x M^{pro} (dimer)	67593.3	67592.0	-1.3	B, C
2x M^{pro} + 1x TKB245	68247.0	68243.7	-3.3	B
2x M^{pro} + 2x TKB245	68900.6	68900.2	-0.4	B
2x M^{pro} + 1x 5h	68169.0	68168.3	-0.7	C
2x M^{pro} + 2x 5h	68744.6	68743.8	-0.8	C

Species of M^{pro}_{E166V}	Theoretical mass	Experimental mass*	Mass error	Fig. 3
M^{pro} (monomer)	33766.7	33765.7	-1.0	A
2x M^{pro} (dimer)	67533.3	67530.9	-2.4	A
2x M^{pro} + 1x Nirmatrelvir	68032.8	68031.8	-1.0	A
2x M^{pro} + 2x Nirmatrelvir	68532.4	68525.0	-7.4	A

Species of M^{pro}_{WT}	Theoretical mass	Experimental mass*	Mass error	Fig. 3
M^{pro} (monomer)	33766.7	33765.4	-1.3	B, C
2x M^{pro} (dimer)	67533.3	67531.4	-1.9	B, C
2x M^{pro} + 1x TKB245	68187.0	68187.8	0.8	B
2x M^{pro} + 2x TKB245	68840.7	68833.5	-7.2	B
2x M^{pro} + 1x 5h	68109.0	68109.0	0.0	C
2x M^{pro} + 2x 5h	68684.7	68683.0	-1.7	C

Species of M^{pro}_{A191T}	Theoretical mass	Experimental mass*	Mass error	Fig. 3
M^{pro} (monomer)	33826.7	33825.7	-1.0	A, C
2x M^{pro} (dimer)	67653.3	67651.4	-1.9	A, C
2x M^{pro} + 1x Nirmatrelvir	68152.9	68153.2	0.3	A
2x M^{pro} + 2x Nirmatrelvir	68652.4	68651.7	-0.7	A
2x M^{pro} + 1x 5h	68229.0	68227.4	-1.6	C
2x M^{pro} + 2x 5h	68804.7	68805.6	0.9	C

Species of M^{pro}_{A191T}	Theoretical mass	Experimental mass*	Mass error	Fig. 3
M^{pro} (monomer)	33826.7	33825.7	-1.0	B
2x M^{pro} (dimer)	67653.3	67651.7	-1.6	B
2x M^{pro} + 1x TKB245	68307.0	68308.3	1.3	B
2x M^{pro} + 2x TKB245	68960.7	68959.3	-1.4	B

Fig. S5. Species of M^{pro} or M^{pro}-compound complexes observed by native MS. Species of M^{pro} or M^{pro}-compound complexes observed in native MS seen in Fig. 3 were identified by the comparison between each deconvoluted mass from measured spectra and corresponding theoretical mass. *Mean values of deconvoluted masses determined using at least three charge states.

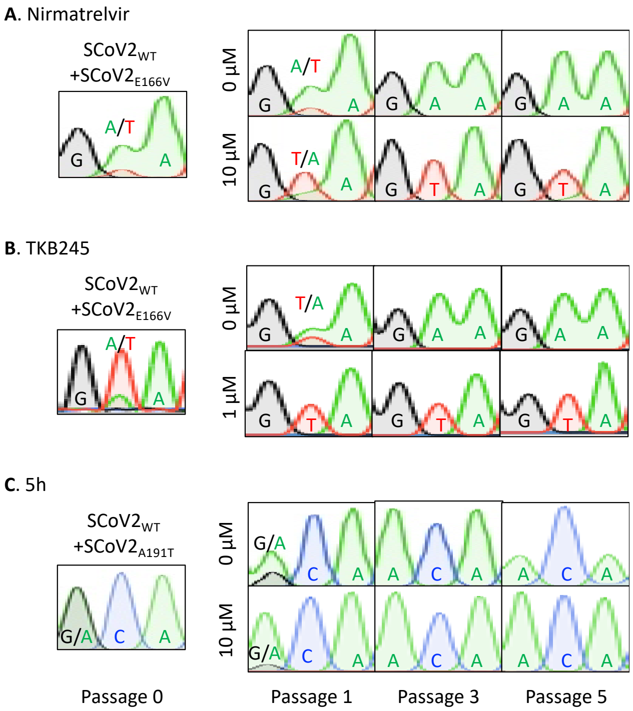

Fig. S6. Electropherogram showing the base changes in the CSRA. Base changes at amino acid positions 166 of M^{pro} in the presence of nirmatrelvir (A)(10 µM) or TKB245 (B)(1 µM) and 191 of M^{pro} in the presence of 5h (C)(10 µM) are shown at passages 0, 1, 3, and 5.

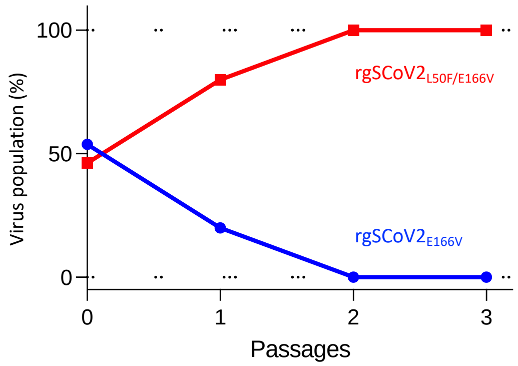

Figure S7. L50F substitution in M^{pro} compensates for the reduced replication fitness of SCoV2$_{E166V}$. Replication profiles of rgSCoV2$_{E166V}$ (blue) and rgSCoV2$_{L50F/E166V}$ (red) were examined in CSRA. Viral RNA extracted from supernatants at the end of each passage was subjected to Sanger sequencing, and the proportions of Leu and Phe at position 50 in M^{pro}s were determined.

Table S1. Susceptibility of M^{P}I-selected SARS-CoV-2 variants against M^{P}Is and profiles of recombinant M^{pro}

Variants	EC_{50}, µM±SD from cell-based assay (fold change from wild-type)						Ki, µM±SD from biochemical assay (fold change from wild-type)		
	VeroE6^{TMPRSS2} cells			Hela$^{hACE2/TMPRSS2}$ cells					
	nirmatrelvir	TKB245	5h	nirmatrelvir	TKB245	5h	nirmatrelvir	TKB245	5h
WT	1.5±0.3 (1)	0.33±0.1 (1)	4.1±1.8 (1)	0.052±0.007 (1)	0.0015±0.001 (1)	0.20±0.09 (1)	0.040±0.008 (1)	0.037±0.001 (1)	0.23±0.004 (1)
E166V	>100 (>67)	13±0.3 (39)	5.4±3.2 (1.3)	29±7 (558)	0.14±0.05 (93)	0.18±0.005 (0.9)	117±3 (2925)	17.1±1.9 (457)	0.69±0.4 (4.1)
A191T	3.9±1.8 (2.6)	0.39±0.2 (1.2)	>100 (>24)	0.042±0.003 (0.81)	0.0017±0.0003 (1.1)	35±0.08 (175)	0.063±0.04 (1.5)	0.181±0.02 (4.8)	3.35±0.04 (15)

Cell-based antiviral assays were conducted using M^{P}I-selected SCoV2 variants. EC_{50} values for each compound were determined using % reduction in viral RNA copy numbers in the no-drug controls and represent averages ± one-standard deviations obtained from at least three independent experiments. Target cells used were VeroE6^{TMPRSS2} and Hela$^{hACE2/TMPRSS2}$ cells. In the enzymatic analyses, recombinant M^{pro} preparations were employed. Fold-changes in parentheses denote the values compared to the control values.

Table S2. Enzymatic characterization of recombinant M^{pro}.

Amino acid substitution	k_{cat}/K_m (fold-change)
WT	286.55 (1)
E166V	2.397 (0.0084)
A191T	115.04 (0.40)

Fold-change of k_{cat}/K_m is defined as a ratio of k_{cat}/K_m for wild-type over k_{cat}/K_m for recombinant M^{pro}_{E166V} or M^{pro}_{A191T}.

Table S3. Antiviral profiles of compounds against recombinant SCoV2 mutants.

Mutants	EC_{50}±SD (μM)		
	nirmatrelvir	TKB245	5h
$rgSCoV2_{WT}$	4.4±0.21	0.78±0.033	13.5±0.58
$rgSCoV2_{E166V}$	>100	6.6±1.1	13.5±0.15
$rgSCoV2_{L50F/E166V}$	>100	11.4±1.4	13.4±0.48

EC_{50} values for each compound were determined using % reduction in viral RNA copy numbers from the no-drug control and represent average and one standard deviation obtained from three independent experiments. The target cells used were VeroE6^{TMPRSS2} cells.

Table S4. Data collection and refinement statistics (molecular replacement)

	M^{pro}_{WT} TKB245 (9ARQ)	M^{pro}_{E166V} TKB245 (9ARS)	M^{pro}_{A191T} TKB245 (9ART)	M^{pro}_{E166V} Apo (8UH8)
Data collection				
Space group	P 21 21	P 1 21 1	P 1	C 1 2 1
Cell dimensions				
a, *b*, *c* (Å)	45.63, 64.00, 105.65	45.93, 53.95, 114.97	45.11, 53.54, 63.86	13.07, 53.76, 44.64
(°)	90.0, 90.0, 90.0	90.0, 100.38, 90.0	114.79, 97.99, 89.99	90 100.911 90
Resolution (Å)	41.92-2.00 (2.03-2.00) *	45.18 - 2.398 (2.51 - 2.4)*	57.3 - 1.49 (1.51 - 1.49)*	48.39 - 1.901 (1.969 - 1.901) *
R_{sym} or R_{merge}	0.114 (0.78)	0.06562 (0.5237)	0.05614 (0.8586)	0.04599 (0.3087)
I / □I	9.30 (0.89)	14.26 (2.26)	6.58 (0.29)	23.64 (2.43)
Completeness (%)	94.3 (63.4)	98.66 (97.13)	92.70 (56.10)	98.09 (96.25)
Redundancy	3.8 (2.3)	3.8 (3.8)	2.0 (2.1)	7.0 (7.2)
Refinement				
Resolution (Å)	41.92 - 2.00	48.69 – 2.40	57.3 - 1.49	55.51 - 1.901
No. reflections	20285 / 1019	21740 / 2641	84234 / 2710	20473 / 2006
R_{work} / R_{free}	0.181 / 0.235	0.178 / 0.222	0.196 / 0.231	0.205 / 0.255
No. atoms	2503	4858	5102	2421
Protein	2426	4739	4729	2365
Ligand/ion	80	90	82	0
Water	91	29	291	45
B-factors	46.51	50.82	29.94	43.09
Protein	29.6	50.90	29.68	43.17
Ligand/ion	32.7	49.58	28.34	
Water	29.2	41.05	34.63	38.78
R.m.s. deviations				
Bond lengths (Å)	0.0103	0.009	0.009	0.013
Bond angles (°)	1.891	1.69	1.58	1.21

*Values in parentheses are for the highest-resolution shell.
